# Design and Comparative Study of a Small-Stroke Energy Harvesting Floor Based on a Multi-Layer Piezoelectric Beam Structure

**DOI:** 10.3390/mi13050736

**Published:** 2022-05-03

**Authors:** Xiang Zhong, Hengyang Wang, Lin Chen, Mingjie Guan

**Affiliations:** 1 Department of Instrumental and Electrical Engineering, Xiamen University, Xiamen 361005, China; zhongxiang@stu.xmu.edu.cn (X.Z.); 35020201151416@stu.xmu.edu.cn (H.W.); llin@xmu.edu.cn (L.C.); 2 Kehua Hengsheng Co., Ltd., Xiamen 361005, China

**Keywords:** piezoelectric, energy harvesting floor, stepping machine, simply supported beam, interdigitated

## Abstract

Recently, research on the energy harvesting floor is attracting more and more attention due to its possible application in the smart house, invasion monitoring, internet of things, etc. This paper introduced a design and comparative study of a small-stroke piezoelectric energy harvesting floor based on a multi-layer piezoelectric beam structure. The multi-layer piezoelectric beams are designed based on simply supported beams in an interdigitated manner. Theoretical analysis is explored to find out the beam number and layer number of the structure. Through this design, the input power from the human footsteps was effectively utilized and transformed into electrical power. The designed piezoelectric energy harvesting floor structure was tested by our designed stepping machine, which can simulate the stepping effect of a walking human on the floor with different parameters such as stepping frequency. Comparative studies of the energy harvester are carried out regarding different stepping frequencies, external circuits, and initial beam shapes. The experimental results showed that the maximum output power of a group of four-layer prototypes was 960.9 µW at a stroke of 4 mm and a step frequency of 0.83 Hz, with the beams connected in parallel.

## 1. Introduction

In recent years, with the development of microelectronics and low-power circuit design, the volume of the wireless sensor and the power consumption have been decreasing. Wireless sensors have been widely used in industry, military, aerospace, medicine, environmental monitoring, and other fields [1,2]. Power of milliwatt level or even microwatt level can now meet the power requirement of a low-power wireless sensor [3]. At present, the power supply of these sensors mainly relies on chemical batteries which have several disadvantages such as limited lifetime, pollution problems, etc. Therefore, it is desirable to harvest the energy from the surrounding environment to provide sustainable power for low-power wireless sensors. There are many kinds of energy in the ambient environment, such as solar [4], wind [5], thermal [6], and kinetic [7] energy. Among them, kinetic energy is ubiquitous in our daily activities. For example, a great amount of kinetic energy can be harvested during human walking [8,9]. In this study, our purpose is to design and fabricate an energy harvesting floor to harvest energy from human footsteps.

At present, several energy harvesting floor structures have been designed. Zhang et al. [10] designed an electromagnetic energy harvester to collect the mechanical energy from human footsteps. However, the magnetic device is usually bulky and a relatively larger stroke is needed. The piezoelectric energy harvester has received more interest in the energy harvesting floor due to its simple structure and small stroke. Isarakorn et al. [11] designed a double-stage piezoelectric energy harvesting floor tile. When actuated by a single simulated footstep, the piezoelectric cantilever beam in it resonated at a frequency of 14.08 Hz. However, the cantilever-beam-based structure had one problem in that a large space was needed for the seismic mass and the free vibration of the beam. Panthongsy et al. [12] fabricated an energy harvesting floor using a piezoelectric frequency up-converting mechanism by magnetic plucking. The results showed a successful frequency up-conversion with a resonant frequency of 10.54 Hz. Nonetheless, the seismic mass of the cantilever beam and the magnetic structure makes the structure bulky.

Piezoelectric simply supported beam structure is another choice for better space utilization. Kim et al. [13] designed a piezoelectric energy harvesting floor with 20 piezoelectrics simply supported beams. The results showed the peak output voltage and current are 42 V and 11 µA, respectively. Our previous work [14] proposed a piezoelectric energy harvesting floor with a force amplifying mechanism and simply supported beam structure. From the experiments, the maximum output power for the piezoelectric beams was tested to be 134.2 µW. To increase the power density in the same floor area, increasing the layers of the piezoelectric elements is a straightforward method. An example is to use the piezoelectric stack [15]. However, the piezoelectric stack has a problem with high stiffness and low strain. This paper proposed a novel energy harvesting floor structure with multi-layer piezoelectric simply supported beams. The structure also utilizes an interdigitated structure.

In the testing of the energy harvesting floor system, usually, human volunteers are arranged to step on the designed floor repeatedly. In Panthongsy et al. [12], the energy harvesting floor tile was placed on a passageway in front of a classroom where 45 students had to walk by and step on it. In Kim et al. [13], experiments were performed by humans with different weights of 60 kg, 65 kg, and 80 kg. In Evans et al. [15], an energy harvester was subjected to both a walking and jogging pedestrian input of a 100 kg human for testing. In Puscasu et al. [16], the energy-harvesting tile was also tested with consecutive steps taken on it. In Tan et al. [17], an adult man walked on the standard block at different frequencies. In Shi et al. [18], the experiment was conducted with the participation of an adult with a weight of 60 kg. In Muñoz et al. [19], experiments were performed by people with different weights of 55 kg, 70 kg, 80 kg, and stepping patterns on the platform in various positions. In Gu et al. [20], when a child (~20 kg), a teenager (~40 kg), and an adult (~60 kg) walked on the energy harvesting floor, different driven forces acted on the energy harvesting floor generating output with different amplitudes. In Wu et al. [21], the output energy was derived from the average value of 10 tests, which were carried out by 10 people. From the above research, the testing procedure by humans is labor-intensive and boring for the volunteers. In addition, as it is very difficult for the volunteers to control the frequency or the force of the footsteps, it is unreasonable to use the testing results from human footsteps for precise analysis and comparative studies.

In order to solve this problem, testing systems were employed in some research. In Panthongsy et al. [12], a motor and a snail cam for simulating the force of human steps were applied. Hwang et al. [22] used a vibration exciter system in the experiments as the exciting source to test the piezoelectric energy harvesting floor. However, the above testing systems are too simple and cannot replicate accurately the force effect during human gait cycles. In this paper, a stepping machine that can simulate the footsteps of humans was designed. Considering most of the energy harvesting floors had the same feature that the upper plate of the floor has a stroke when people step on it, the target of the designed stepping machine was to replicate the displacement profile of the upper plate of the floor structure instead of the force profile from the foot to the floor plate. By use of cyclical footsteps from the stepping machine, comparative studies regarding the parameters of the piezoelectric energy harvesting floor structure were carried out.

This paper is organized as follows. In Section 2, the design, analysis, and fabrication of the piezoelectric energy harvesting floor are described. In Section 3, the development of the stepping machine system is described. In Section 4, comparative studies of the energy harvesting floor with the help of the stepping machine are given. Finally, conclusions and discussions are presented in Section 5.

## 2. Design, Analysis, and Fabrication of the Energy Harvester

**Figure 1:**
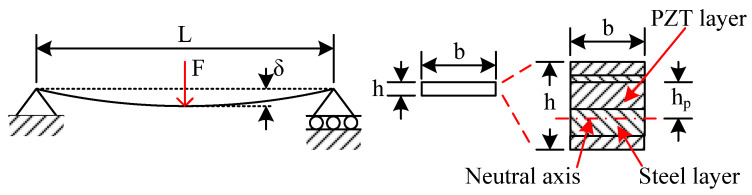
Schematic of the simply supported piezoelectric beam structure with a cross-sectional area (not to scale).

To better utilize the power from the footsteps and convert them into electrical power by piezoelectric elements, an efficient structure should be designed. In this design, simply supported piezoelectric beams are used. Assuming the beam has a length L, a width b, and a total height of h, as shown in Figure 1.

The relation between the deflection of the beam *δ* and the applied concentrated force *F* at the middle point of the beam is:

*δ = FL^3^/(48E_b_I_b_)*, (1)

where *E_b_I_b_* is the flexural rigidity of the beam, *E_b_* is the equivalent Young’s modulus of the beam, and *I_b_* is the equivalent moment of the inertia of the cross-sectional area. In this design, to better protect the piezoelectric element, a piezoelectric product modeled PPA1001 (Piezo Systems, Inc., Woburn, MA, USA) is used as the piezoelectric beam substrate. It has five layers: polyester, copper, PZT, stainless steel 304, and polyimide, respectively. The thicknesses of the layers are 0.05 mm, 0.03 mm, 0.15 mm, 0.15 mm, and 0.03 mm, respectively. Young’s moduli of the layers are 3.6, 110, 51, 193, and 4.1 GPa, respectively. The PZT layer is protected by other layers, which significantly increases the robustness of the PZT element. The length L is 46 mm. The width b is 23.4 mm. The total height h is 0.41 mm. The permitted deflection of the beam is 2 mm. As Young’s moduli of these five layers are different, the neutral plane of the beam when deformed should firstly be found out. Through calculation, the neutral plane is about 0.01 mm from the PZT layer, as shown in Figure 1. Therefore, the maximum distance of the piezoelectric layer to the neutral plane *h_p_* is 0.16 mm. For the multi-layer cross-section, the maximum strain of the PZT layer is related to the applied force F by

*ε_max_ = FLh_p_/(4E_b_I_b_)*, (2)

From Equations (1) and (2), the relation of strain and the deflection is

*ε_max_ = 12h_p_δ/L^2^*, (3)

To better utilize the electromechanical transformability of the piezoelectric element, the strain of the piezoelectric element should be near the permitted strain while it is deformed at the deflection limit.

From Equation (1), the permitted force F is calculated as about 8.282 N. When a human with a weight *m* steps on a floor plate, the applied force *P* to the plate can reach 130% of body weight from a typical human gait [23]. Therefore, to better utilize the force from the human footsteps, it is found that the number of the piezoelectric beams can be

*N = P/F = 62.4mgE_b_I_b_/(δL^3^)*, (4)

Considering a human weighing 68 kg, the number *N* will be 104.6. For a floor plate that covers a foot size of 300 mm × 100 mm, the plate can include at most 27 beams with an area of 46 mm × 23.4 mm in one layer. Therefore, one layer of piezoelectric beam is not enough. At least four layers of piezoelectric beams are needed.

**Figure 2:**
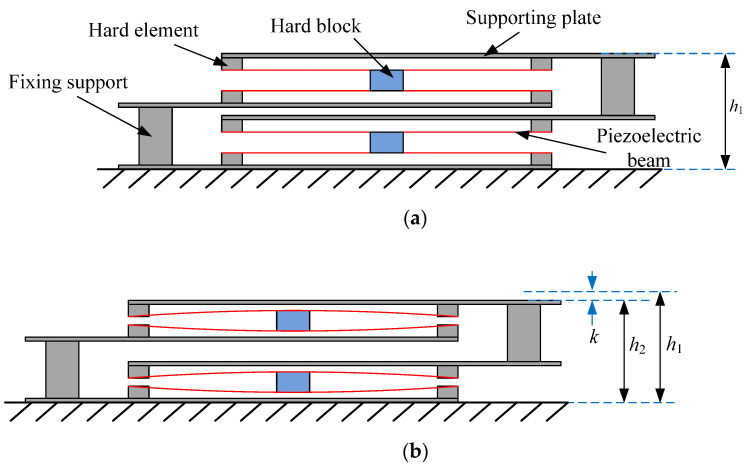
Design and working principle of the multi-layer interdigitated piezoelectric beam structure: (**a**) before stepped; (**b**) after stepped.

Based on the above analysis, the designed multi-layer piezoelectric harvesting floor structure is shown in Figure 2a. As the maximum deflection is 2 mm and the stroke of the floor plate is targeted as 4 mm, an interdigitated structure is applied. The interdigitated structure can let the deflection equal half of the stroke of the plate. In the interdigitated structure, the piezoelectric harvesting floor structure is divided into two parts: the upper part and the lower part. The upper and lower parts both have two layers of piezoelectric beams. Therefore, there are four layers of piezoelectric beams in total. For each beam, two hard elements were bonded to the supporting plate, and then the piezoelectric beam was adhered to the hard elements to form a simply supported beam. Each beam from the upper structure and the adjacent beam from the lower structure formed a couple of beams. Between a couple of beams was a hard block for passing force, which is bonded in the middle of the beams. As shown in Figure 2b, when the top supporting plate is stepped down, the upper structure descends, and the stroke is denoted as *k*. The deflections of the beams will be *k*/2. Voltages will be generated from the deformed piezoelectric beams.

**Figure 3:**
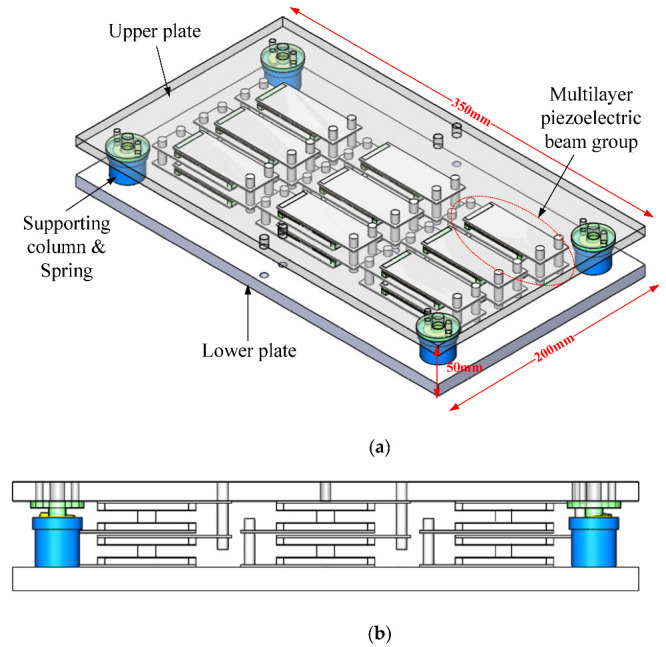
Structure of the energy harvesting floor: (**a**) three-dimensional view; (**b**) front view.

Based on the working principle of the multi-layer piezoelectric beams, a piezoelectric energy harvesting floor structure was designed. The whole structure, built in a 3D model, is shown in Figure 3. The overall size of the floor structure is 350 mm × 200 mm × 50 mm. For demonstration, nine groups of multi-layer piezoelectric beam structures are placed inside the floor structure. Four supporting columns and springs were installed at four corners of the bottom plate to support the upper plate and limit the stroke of the upper plate to protect the piezoelectric beams. For each simply supported beam, two hard elements of 3 mm height are used.

**Figure 4:**
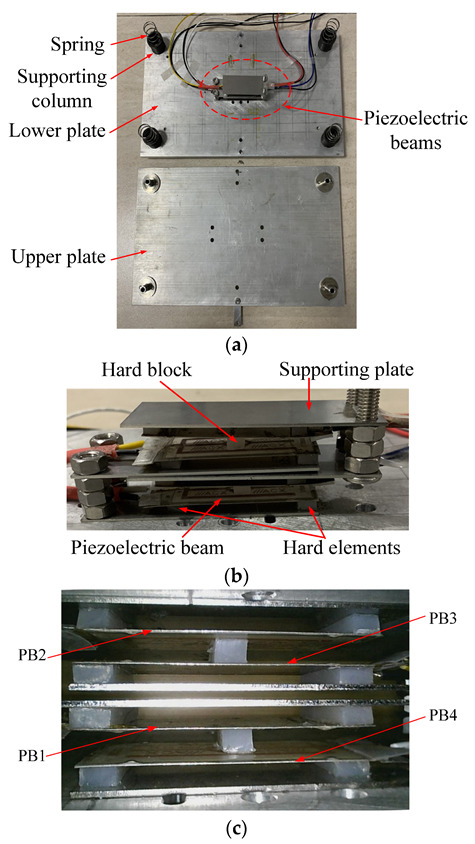
Prototype of energy harvesting floor structure: (**a**) whole view; (**b**) close-view of piezoelectric beams; (**c**) side-view of piezoelectric beams.

The prototype of the piezoelectric harvesting floor with one group of piezoelectric beams is shown in Figure 4, a close view of the multi-layer piezoelectric beams is shown in Figure 4b, and a side view of the piezoelectric beams is shown in the Figure 4c. There are four piezoelectric beams inside one group of beams, marked as PB1~4. The capacitances of the four piezoelectric elements in the beams are 90 nF, 93 nF, 95nF, and 96 nF, respectively. The thickness of the hard block was set as 4 mm. The piezoelectric beams were all flat and non-deformed in the initial state. Some properties of the plates and the piezoelectric beams are shown in Table 1.

## 3. Development of the Stepping Machine

To test the piezoelectric floor, instead of stepping on the floor with a human volunteer, a stepping machine system was developed which can exert a stepping force on the floor to simulate a human stepping on it. Through the stepping machine, the exerted force on the floor during each step can be controlled.

### 3.1. Design of Stepping Machine

**Figure 5:**
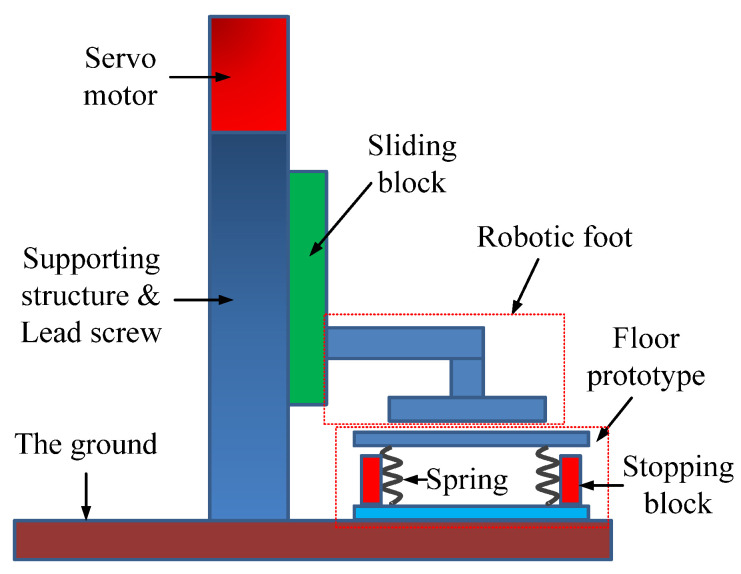
Structure of the stepping machine (side view).

The stepping machine was designed as shown in Figure 5. It mainly includes five parts: servo motor, lead screw, sliding block, and robotic foot. When the motor rotates, the rod of the lead screw will rotate through the coupling. Then the lead screw will transform the rotation into a linear motion and make the sliding block move up/down. Meanwhile, the robotic foot connected to the sliding block will move up/down and step on/off the floor. The floor structure can be simplified as a mass-spring system. While the floor was stepped down by the machine, the upper plate would move downward until it was limited by the stopping block. After the stepping, the upper plate will return to its initial position.

**Figure 6:**
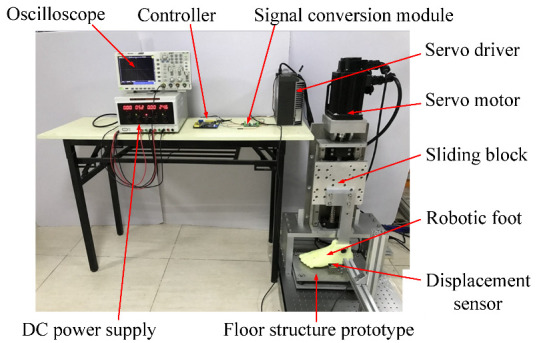
Prototype of stepping machine system (front view) and the testing setup.

The prototype of the stepping machine system was built as shown in Figure 6. The stepping machine was dimensioned 60 cm × 80 cm × 120 cm. For the lead screw, the rod is made of Aluminum 6063; the helical pitch is 10 mm with a resolution of 0.03 mm. To meet the requirement of the stepping speed and force of the stepping machine, the motor should output a rotation speed that is larger than 1200 r/min and a torque larger than 1.2 Nm. A synchronous AC motor modeled as 110st-M06030 (MIGE Motor Co., Ltd., Hangzhou, China) is used, which can offer a rated rotating speed of 3000 r/min, and a rated load torque of 1.2 Nm. The servo driver is modeled DO-1000C/50A (MIGE Motor Co., Ltd., Hangzhou, China). The resolution of the servo motor can reach 0.003°. An STM32 controller is used to control the motor. A signal conversion module is used to convert the electrical signal from the controller to the servo system.

### 3.2. Control of the Setpping Machnie

**Figure 7:**
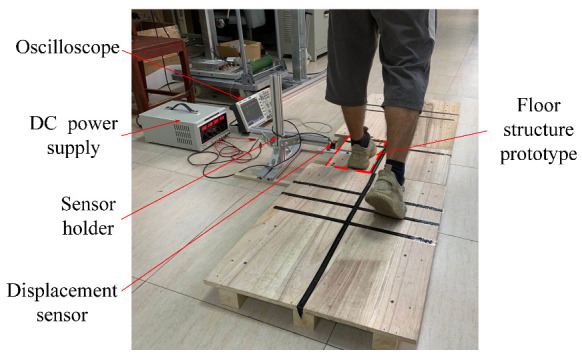
Experimental setup to acquire the displacement data from human footsteps.

**Figure 8:**
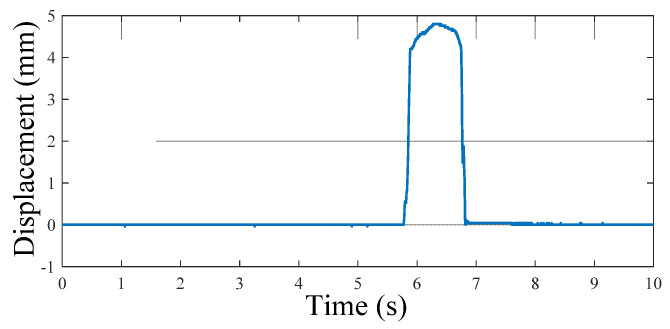
Displacement curve of the upper plate of the floor structure.

To control the stepping machine, at first, the displacement profile from a human footstep over the floor structure is analyzed. The experimental setup to acquire the displacement data from human footsteps was constructed as shown in Figure 7. A pedestrian road was set up with a wooden board. The floor structure prototype was placed on the left side of the wooden road. The displacement of the upper plate of the floor structure was measured by a laser displacement sensor, modeled HG-C1030 (Panasonic Co., Osaka, Japan). The displacement sensor has a resolution of 10 µm and a measuring range of 10 mm. Then, the output voltage was sent to the oscilloscope, (NDS104E, OWON, Zhangzhou, China). An example of the displacement curve from the measuring system is shown in Figure 8. The displacement curve is sent to the controller of the stepping machine.

**Figure 9:**
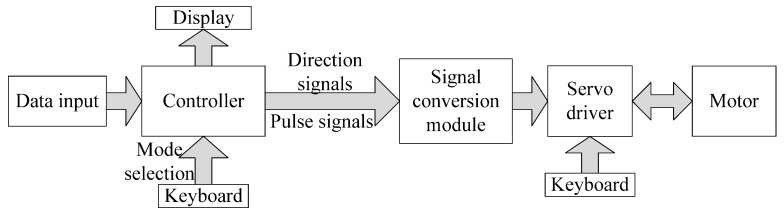
Hardware framework of the stepping machine.

The hardware framework of the stepping machine is shown in Figure 9. The controller of the system is modeled STM32F103. The programming software is Keil uVision 5. The original footstep displacement data are sent to the controller. The controller works on them and converts them into speed data for the motor. The timers of the controller are used to generate pulse signals and direction signals for the motor driver. The controller works on the timer interruption function mode by adjusting the values of “Auto Reload Register” and “Pre-Scaler Register”. Then, the direction signals and pulse signals with amplitudes of 0~3.3 V are output and sent to the signal conversion module. Through the signal conversion, the amplitudes of the signals are transformed to 0~5 V for the servo driver. The servo driver makes the motor rotate accordingly.

### 3.3. Validation of the Stepping Machine

**Figure 10:**
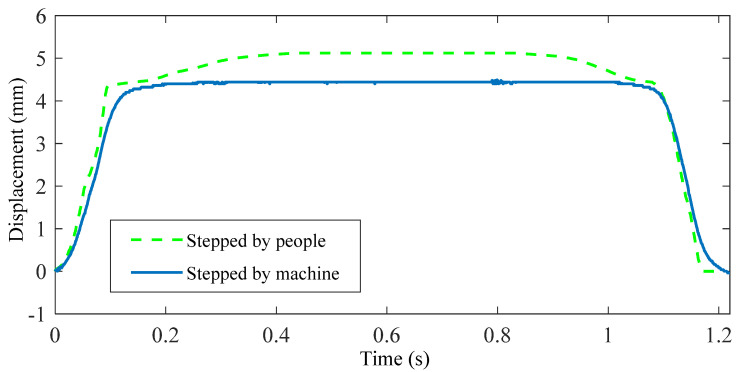
Comparison of the original displacement curve and output displacement curves.

The testing setup of the stepping machine was shown in Figure 6. The displacement data from human footsteps was firstly measured and sent to the controller. Then, the displacement from the stepping machine system is measured by the laser displacement sensor. In the experiment, the displacement from a volunteer’s footstep was shown in the green dash line in Figure 10, and the displacement curve driven by the machine is shown in the blue solid line for comparison. From the results, it is demonstrated that the stepping machine system can well replicate the displacement profile stepped by humans. The rising time, rising slope, falling time, and falling slope are very close to each other. However, the maximum displacement from the human is larger than the displacement by the machine. One reason is that the machine system only drives the robotic foot in one vertical degree whereas the interaction force of the human footstep on the floor during walking is much more complicated. A two-degree stepping machine system is still under research. It should be noted that if the walking person has another weight, the input displacement data may be different. Moreover, if one person walks with different gaits, the input displacement data would be different too. Therefore, the function of the stepping machine is to output a cyclical stepping profile according to different inputs, which may come from different persons or different gaits.

## 4. Comparative Studies of the Energy Harvesting Floor

**Figure 11:**
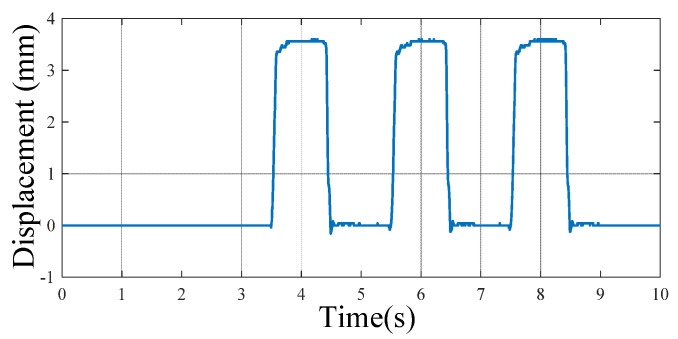
Displacement output of the stepping machine system with 3 continuous steps.

Through programming the stepping machine, consecutive steps with different frequencies can be output. An example of three consecutive steps by the machine is shown in Figure 11, with a frequency of 0.5 Hz. The comparative studies of the energy harvesting floor were carried out by use of the stepping machine.

### 4.1. Output Voltages with Different External Circuits

**Figure 12:**
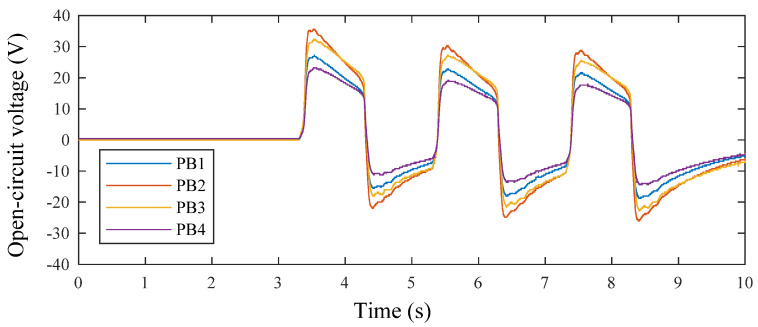
The open-circuit voltages of piezoelectric elements.

In the first experiment, the piezoelectric energy harvesting floor structure was tested with 3 consecutive steps at the stepping frequency of 0.5 Hz and the output voltages of the piezoelectric elements were measured. The open-circuit voltages of the tested four piezoelectric beams were recorded individually as shown in Figure 12. As can be seen, four voltage curves were shown. The highest voltage amplitude comes from the piezoelectric beam “PB2”; the second from “PB3”; the third from “PB1”; and the lowest from”PB4”. The four curves are not exactly the same mainly because of the difference between the piezoelectric elements themselves and installation conditions. From the voltage curve of “PB2”, the peak values of the three cycles were 35.6 V, 30.4 V, and 28.8 V, respectively. There are slow decreases of the voltage after the peak points in each step, which comes from dielectric losses in the piezoelectric materials. It can also be seen that the peak value decreases. This phenomenon may be due to the charge accumulation on the piezoelectric elements.

**Figure 13:**
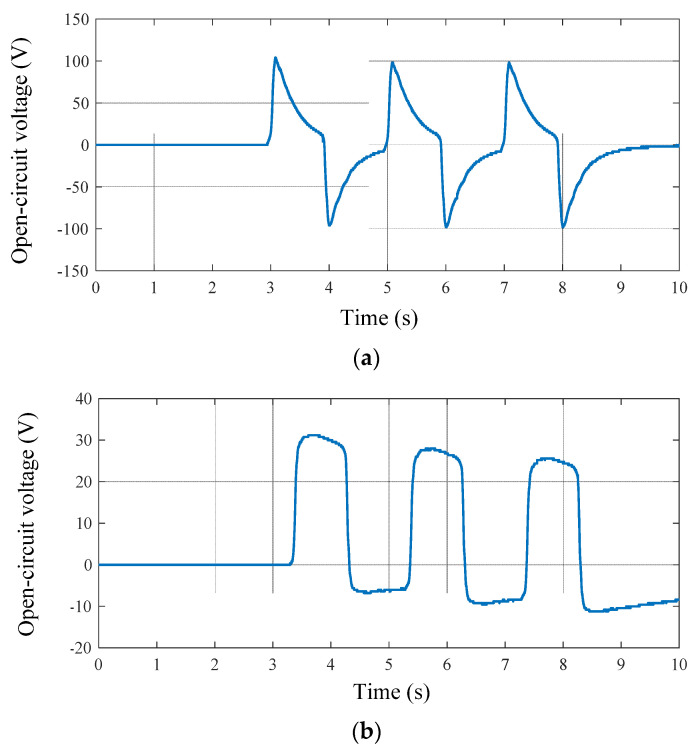
Open-circuit voltages of piezoelectric elements in series and in parallel: (**a**) in series; (**b**) in parallel.

In a piezoelectric element array, piezoelectric elements are usually connected in series, in parallel, or in a compound connection to output power. In the next experiment, the output voltage and power with different connection manners are compared. Still, with a stepping frequency of 0.5 Hz, the open-circuit voltages of the four piezoelectric elements connected in series and in parallel are compared in Figure 13a,b, respectively. It can be seen that the waveform in Figure 13a was steep and the maximum peak-to-peak voltage was 104.0 V. On the other hand, the waveform in Figure 13b was blunt and the maximum peak-to-peak voltage was 32.0 V. Meanwhile, the peak values of the waveform in Figure 13b declined obviously. The reason for the difference between these two waveforms may be that the internal capacitance is decreased in series and increased in parallel, and the time constant is much smaller in series than that in parallel. Therefore, the discharge process in series was much faster than that in parallel.

### 4.2. Output Power under Different Frequencies and External Loads

As shown in Figure 13, the open-circuit voltage curves of the piezoelectric elements in series and in parallel are not sinusoid curves either. Experiments were explored to compare the output power of these two circuit configurations. In this experiment, three stepping frequencies were applied as 0.5 Hz, 0.67 Hz, and 0.83 Hz, respectively. additionally, 16 external load resistances ranging from 20 kΩ to 4 MΩ were used. The output power from piezoelectric elements can be written as

(5) $$\left\{ \begin{array}{l} P_{O}(t)=\frac{U_{O}{}^{2}(t)}{R_{O}^{}}\\ P_{ave}=\frac{1}{T}\int_{0}^{T}P_{O}(t)dt \end{array}\right.$$

where *R_O_* is of the external load; *U_O_*(*t*) is the output voltage across the external load; *P_O_*(*t*) is the instantaneous power of the external load; *P_ave_* is the average output power; *T* is the period of a stepping cycle.

**Figure 14:**
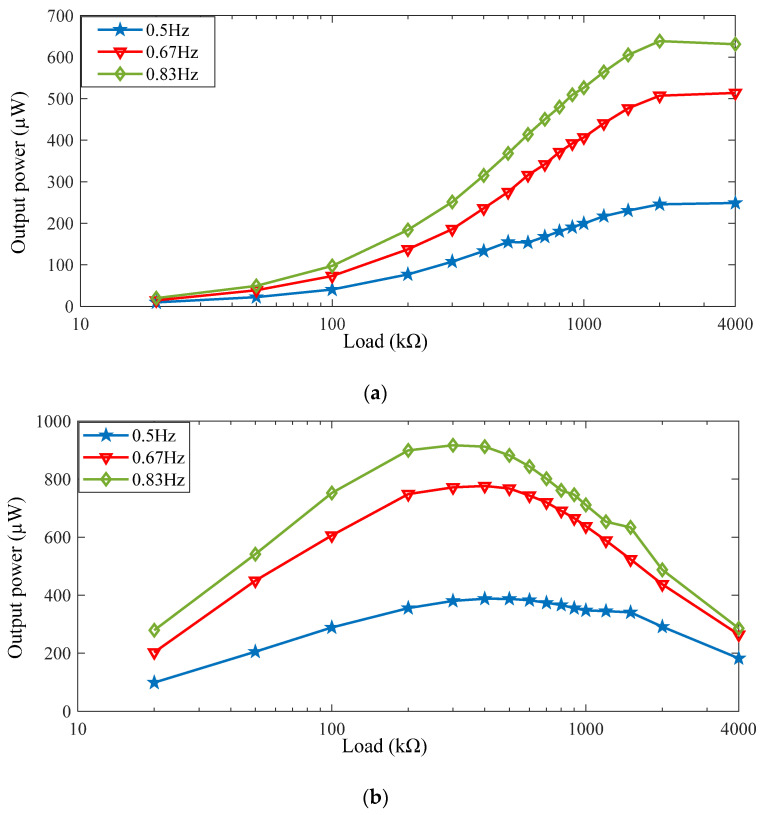
Output power of piezoelectric elements with different loads and step frequencies: (**a**) four piezoelectric elements in series; (**b**) four piezoelectric elements in parallel.

Through the experiments, the relationship between output power and stepping frequency was studied. The output power under different frequencies and different loads were shown in Figure 14. It can be seen that the output power increases apparently with the frequency. When the four piezoelectric elements were connected in series, as shown in Figure 14a, the maximum output power was 638.4 µW at a frequency of 0.83 Hz when the external load was 2 MΩ. When the four piezoelectric elements were connected in parallel, as shown in Figure 14b, the maximum output power was 916.9 µW at a frequency of 0.83 Hz when the external load was 300 kΩ. The results indicate that the maximum output power can be acquired when the piezoelectric elements are connected in parallel other than in series. It is noticeable that the equivalent piezoelectric capacitance of the four elements is reduced in series connection, while in parallel connection, the equivalent capacitance of the four elements is increased. The optimal resistance maximizing the harvested power is proportional to 1/*C_p_*, with *C_p_* being the equivalent capacitance of the piezoelectric elements. Therefore, because of its larger equivalent capacitance, the optimal resistance with the parallel connection is lower than with the series connection. The power density regarding the area of the piezoelectric harvesting floor structure can reach 37.28 μW/cm^2^.

### 4.3. Output Power with Different Initial Beam Shapes

**Figure 15:**
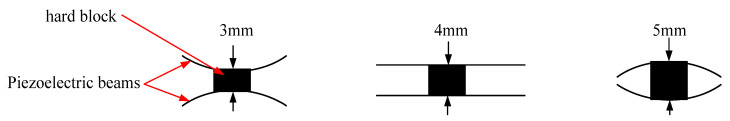
The initial piezoelectric beam shapes with different hard block thicknesses.

Many factors will influence the output power of the piezoelectric energy harvesting floor structure. In this study, the initial piezoelectric beam shape is considered. The initial piezoelectric beam shape can be adjusted by hard blocks of different thicknesses. To study the output power with different initial beam shapes, experiments were conducted with hard block thicknesses of 3 mm, 4 mm, and 5 mm. The stepping frequency was set as 0.83 Hz and the piezoelectric elements were connected in parallel. The initial beam shapes were obviously different as shown in Figure 15. The piezoelectric beams were non-deformed when the block thickness was 4 mm and was deformed when the thickness was 3 mm and 5 mm.

**Figure 16:**
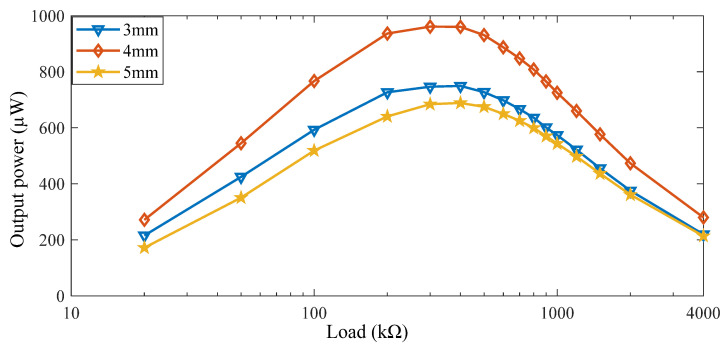
The output power under different hard block thicknesses.

By use of the stepping machine and different external loads, the experimental results were illustrated in Figure 16. The maximum output power was 749.5 µW, 960.9 µW, and 684.2 µW, with thicknesses of 3 mm, 4 mm, and 5 mm, respectively. In addition, the optimal external loads are all about 400 kΩ. It can be found that the output power was the highest when the thickness was 4 mm. That indicates it is better to make the piezoelectric beams in non-deformed states at the initial condition.

## 5. Conclusions and Discussions

The design of a multi-layer piezoelectric energy harvesting floor structure was proposed in this paper. The simply supported beam structure and interdigitated structure were adopted in the design. The interdigitated structure can reduce the deflection of the beam to half of the stroke. In order to efficiently utilize the power from the footsteps, beam number and layer number were theoretically analyzed. In this design, the stroke is 4 mm and four layers were applied. From the theoretical equations, if a smaller stroke (for example, 2 mm) is applied, the beam length, beam height, beam number, and beam layer number could be differently designed.

In order to perform the comparative studies, a stepping machine that can apply a human-like stepping effect on the floor was designed and applied in the research. With the help of the stepping machine, the performances of the designed piezoelectric structure were studied with different stepping frequencies, external circuits, and initial beam shapes. To conclude, with the increase in the stepping frequency, the output power apparently increased. Additionally, the piezoelectric elements connected in parallel can output more power than those connected in series. When the four piezoelectric elements are connected in parallel, the maximum output power can reach 960.9 µW at the frequency of 0.83 Hz when the beam is in a non-deformed shape initially.

The designed energy harvesting floor structure demonstrates the potential of harvesting energy from human footsteps. Taking a wireless sensor node [24] for example, the output power generated from our prototype is enough to make it work. A traditional rectifier bridge can be applied to rectify the voltage for the sensor nodes. More efficient rectifier interfaces [25] can be applied to improve the rectifying efficiency. The designed stepping machine shows the convenience and efficiency in testing the harvesting floor and comparative study. In future work, a multi-degree stepping machine can be studied to improve the performance of testing. The energy conversion structure used in this paper is based on a non-resonant scheme. The advantages and disadvantages of the resonant structure and non-resonant structure for energy conversion will be compared in future work. The fatigue, scalability, and repeatability issues of the designed harvesters will also be studied in future work.

## Figures and Tables

**Table 1:** Properties of the plates and the piezoelectric beams.

Properties	Upper Plate	Lower Plate	PPA-1001
Material	aluminum alloy 5056		PZT-5J
Density (Kg/m^3^)	2660		7800
Young’s modulus (GPa)	72		51
Poisson’s ratio	0.33		0.31
Tensile yield strength (MPa)	290		210
Dimension (mm)	350 × 200 × 10		46 × 23.4 × 0.15
